# Supplementary material for: Dynamic encoding of temperature in the central circadian circuit coordinates physiological activities
Source: Nat Commun. 2024 Apr 2;15:2834. doi: 10.1038/s41467-024-47278-5 (PMC10987497; doi:10.1038/s41467-024-47278-5)
Supplement: Supplementary file 1 — Supplementary Information [file 41467_2024_47278_MOESM1_ESM.pdf]

Dynamic encoding of temperature in the central circadian circuit coordinates physiological activities

Hailiang Li [1, 2, 3], Zhiyi Li [1, 2, 3], Xin Yuan [1, 2, 3], Yue Tian [1, 2, 3], Wenjing Ye [1, 2, 3], Pengyu Zeng [1, 2, 3], Xiao-Ming Li [2, 3, 4] and Fang Guo [1, 2, 3, *]

[1] Department of Neurobiology, Department of Neurology of Sir Run Run Shaw Hospital and School of Brain Science and Brain Medicine, Zhejiang University School of Medicine, Hangzhou, 310058, China.

[2] MOE Frontier Science Center for Brain Research and Brain-Machine Integration, State Key Laboratory of Brain-machine Intelligence, Zhejiang University, 1369 West Wenyi Road, Hangzhou 311121, China.

[3] NHC and CAMS Key Laboratory of Medical Neurobiology, Zhejiang University, Hangzhou 310058, China.

[4] Department of Neurobiology and Department of Psychiatry of the Second Affiliated Hospital, Zhejiang University School of Medicine, Hangzhou, 310058, China.

* Correspondence: gfang@zju.edu.cn (F.G.)

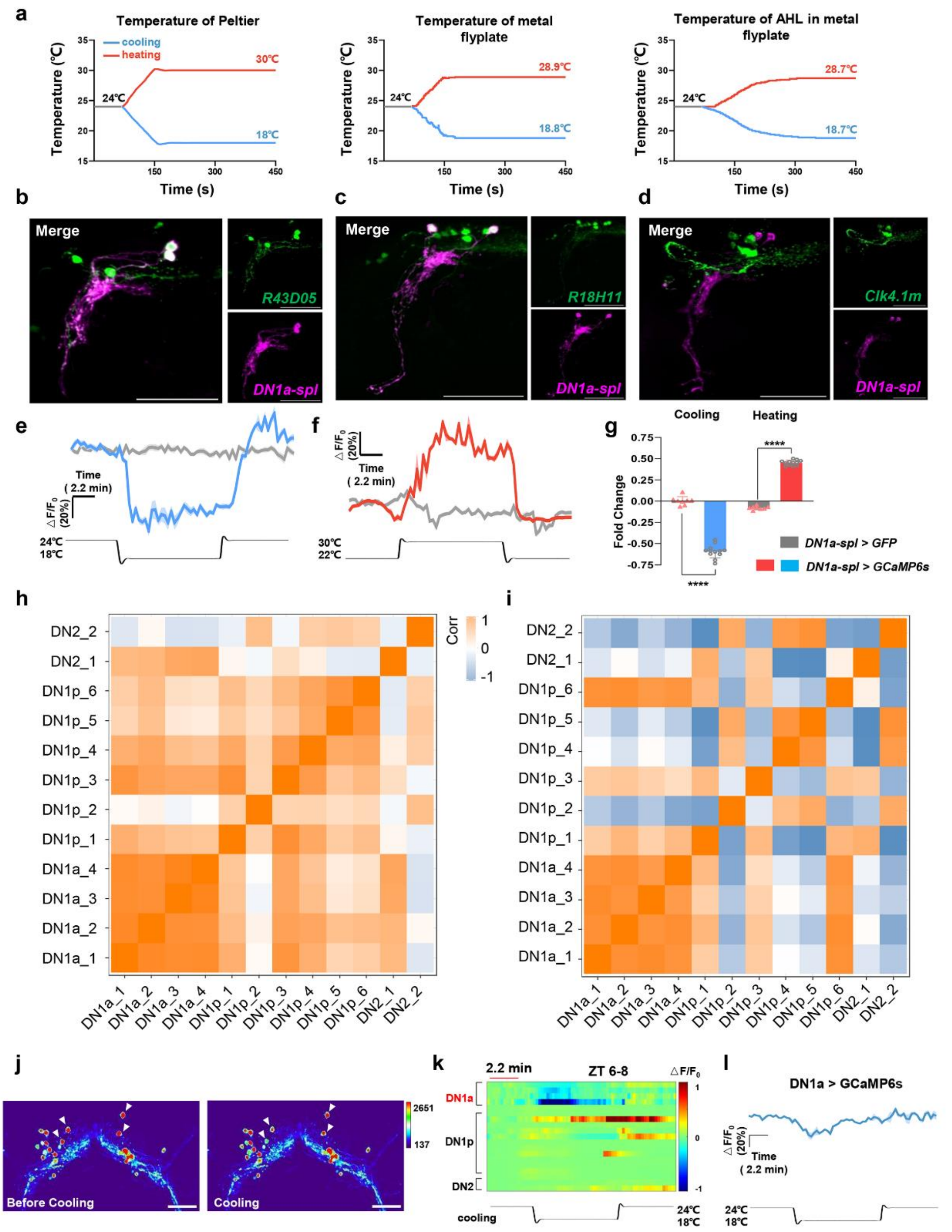

Supplementary Fig. 1: Temperature calibration for accurate control of experimental setup and morphological and functional characterization of DN1as.

a Measured temperature changes of the Peltier, of the metal flyplate, and of the AHL in flyplate throughout live GCaMP imaging. The Peltier element set-point was changed from 24°C to 30°C (red line) and from 24°Cto 18°C (blue line), respectively, for heating and cooling stimulus (see Methods for details).

b-d Double staining of GFP and RFP in *R43D05-LexA > LexAop-GFP, DN1a-spl > UAS-tdTomato* brain (B), in *R18H11-LexA > LexAop-GFP, DN1a-spl > UAS-tdTomato* brain (**c**), in *Clk4.1m-LexA > LexAop-GFP, DN1a-spl > UAS-tdTomato* brain (**d**). Scale bars, 50 µm.

e, f The representative GCaMP (blue, n = 12) and GFP (grey, n = 6) traces ($\Delta F/F_0 \pm$ SEM) of DN1as in response to cooling (**e**) and heating (**f**).

g Quantification of the relative fold change of calcium activities of DN1as in (**e-f**) Data are presented as mean $\Delta F/F_0$ (%) ± SEM in the histogram; n=12, 2-tailed *t*-test; ns: no significant difference; $^{**}p < 0.01$, $^{***}p < 0.001$, left panel: p= 2.25754E-13; right panel: p= 1.51998E-19.

h-i Pairwise correlation matrices of circadian neurons in response to cooling (**h**) and heating (**i**). **h** related to **Fig. 1d**. **i** related to **Fig. 1g**.

j Representative pseudocolor images of calcium responses of *Clk856-GAL4 > UAS-GCaMP6s* flies before and after cooling (**left**) and heating (**right**). white arrow indicates DN1as.

k Heat map of the temporal calcium activities of circadian neurons to cooling (24-18°C). The temperature change was labeled bottom pattern.

l The representative GCaMP traces ($\Delta F/F_0 \pm$ SEM) of DN1as in response to cooling (blue, n = 7, **j**).

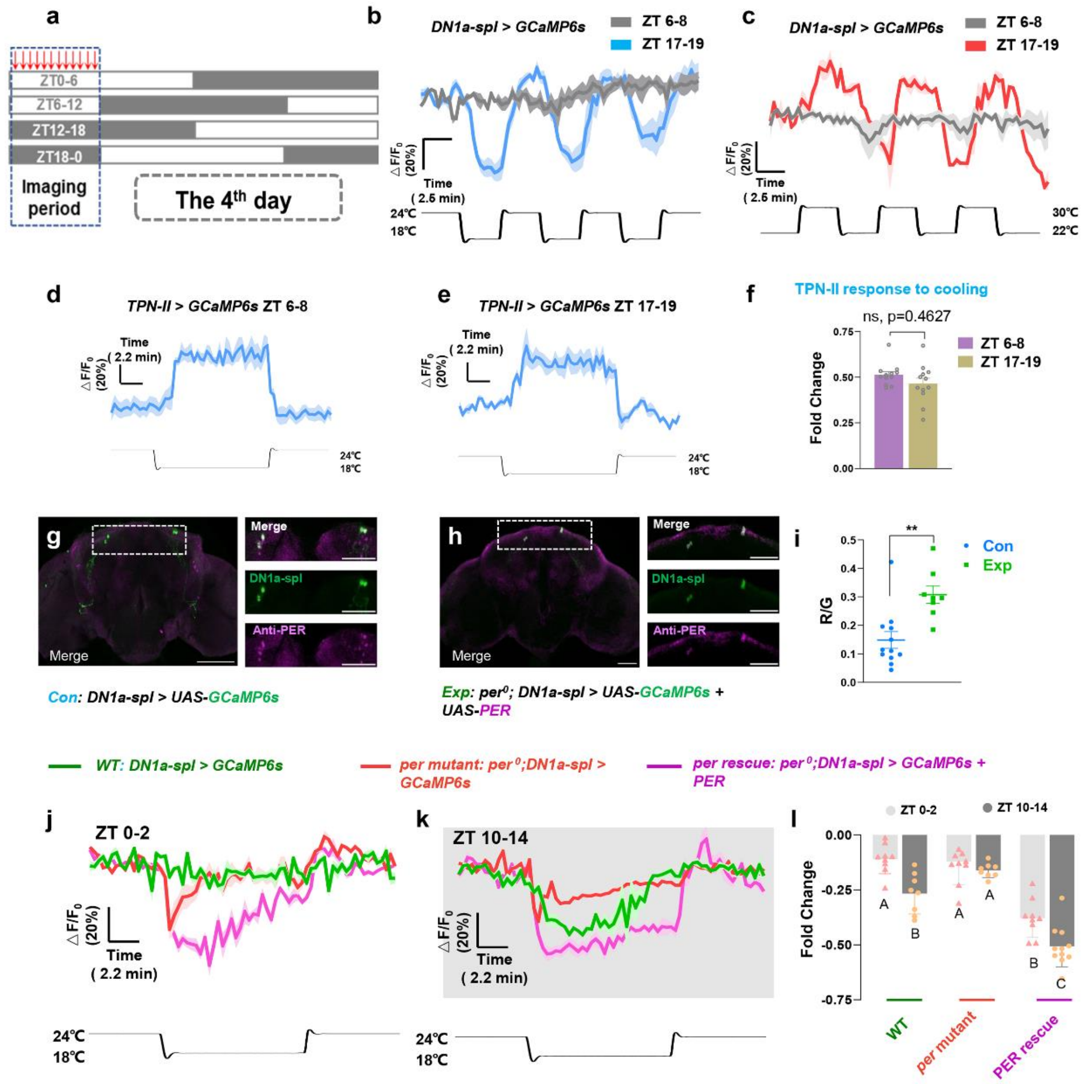

Supplementary Fig. 2: Analyzing the response of both circadian and non-circadian TPN-IIs to temperature changes.

a Schematic diagram of the entire procedure for the long-term in vivo calcium imaging: flies were divided into four groups, each of which underwent a 3-day LD entrainment with a 6-hour shift prior to the imaging period.

b, c DN1as respond reliably to repeated cold (**b**) or hot (**c**) stimuli during daytime (grey, n=8) and night (blue or red, n=8).

d-e The representative GCaMP traces ($\Delta F/F_0 \pm$ SEM) of TPN-IIs in response to cooling (blue, n=8) at ZT6-8 (**d**) and ZT17-19 (**e**).

f Quantification of the relative fold change of calcium activities of DN1as in (**d-e**). Data are presented as mean $\Delta F/F_0$ (%) ± SEM in the histogram; 2-tailed *t*-test; ns: no significant difference. n=8.

g-h PER staining in the brains of wild-type flies (left) and DN1a-specific PER-rescued flies (right). Scale bars, 50 μm.

i Quantification of PER level in DN1as (**g-h**, n = 6). Data are presented as average fluorescence of PER ± SEM in the histogram; 2-tailed *t*-test; ns: no significant difference; $^{**}p < 0.01$, p=3.35005E-06.

j, k The representative GCaMP traces ($\Delta F/F_0$ ± SEM) of DN1as in response to cooling in flies at ZT 0-2 (**j**) and ZT 10-14 (**k**). green curve: wild type; red curve: *per* mutant; purple curve: PER rescue. Dark shades indicate lights off (night); n = 6.

l Quantification of the relative fold change of calcium activities of DN1as in (**j-k**). Data are presented as mean in $\Delta F/F_0$ (%) ± SEM in the histogram; one-way ANOVA; Tukey's honest significance difference test to assess the statistical significance of the mean values within each experimental group. The letters A, B and C above the histograms denote significantly different means within each of the two groups, p<0.05. Specific p-values corresponding to this figure are reported in the Source Data. n=6.

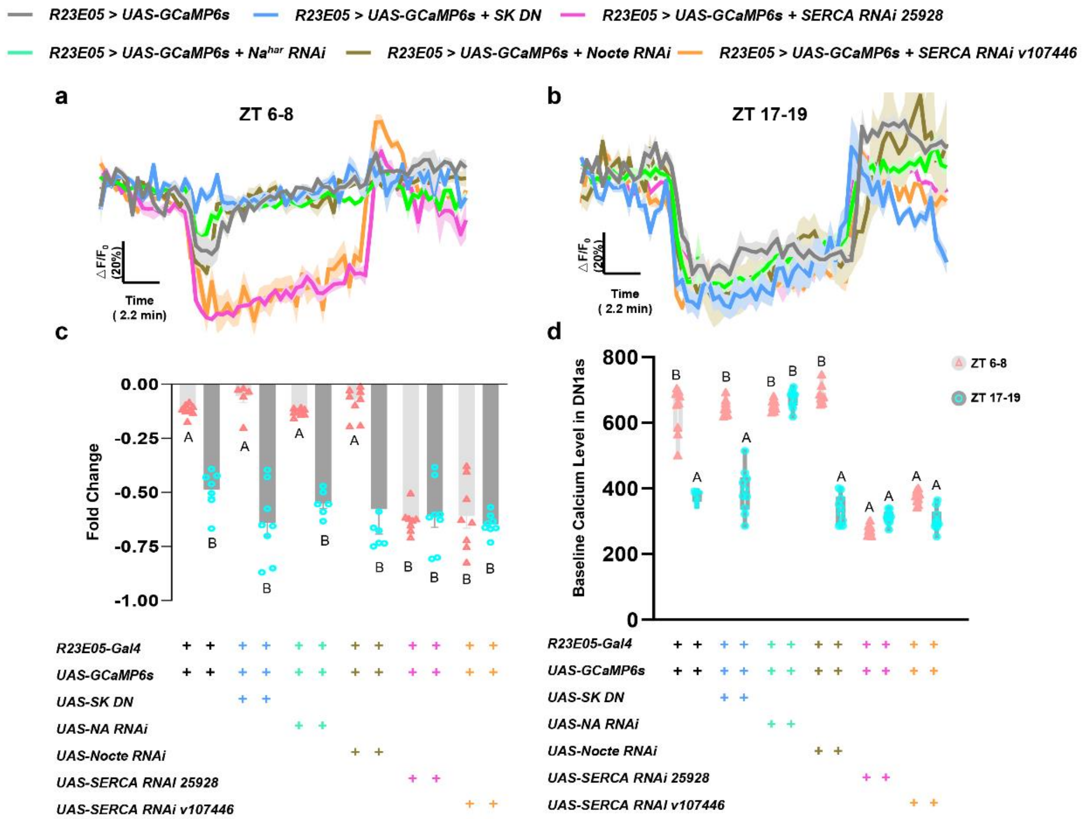

Supplementary Fig. 3: The circadian clock modulates cold-induced diurnal calcium response via SERCA.

a, b The representative GCaMP traces ($\Delta F/F_0 \pm$ SEM) of DN1as in response to cooling in flies at ZT 6-8 (**a**) and ZT 17-19 (**b**). green curve: wild type; red curve: *per* mutant; purple curve: PER rescue. Dark shades indicate lights off (night); n = 6.

c, d (**c**): Quantification of the relative fold change of calcium activities of DN1as in (**a-b**). (**d**): Basal GCaMP6s signals in DN1as at two circadian time points in (**a-b**). Data are presented as mean in $\Delta F/F_0$ (%) ± SEM in the histogram; one-way ANOVA; Tukey's honest significance difference test to assess the statistical significance of the mean values within each experimental group. In the provided histograms labeled A, B, C and D, the use of the same letter denotes the absence of a significant difference between the two groups, whereas differing letters signify a $p<0.05$, indicating a significant distinction between the groups. The baseline calcium level represents the spontaneous calcium level of DN1a before temperature fluctuations. n=6. Specific p-values corresponding to this figure are reported in the Source Data.

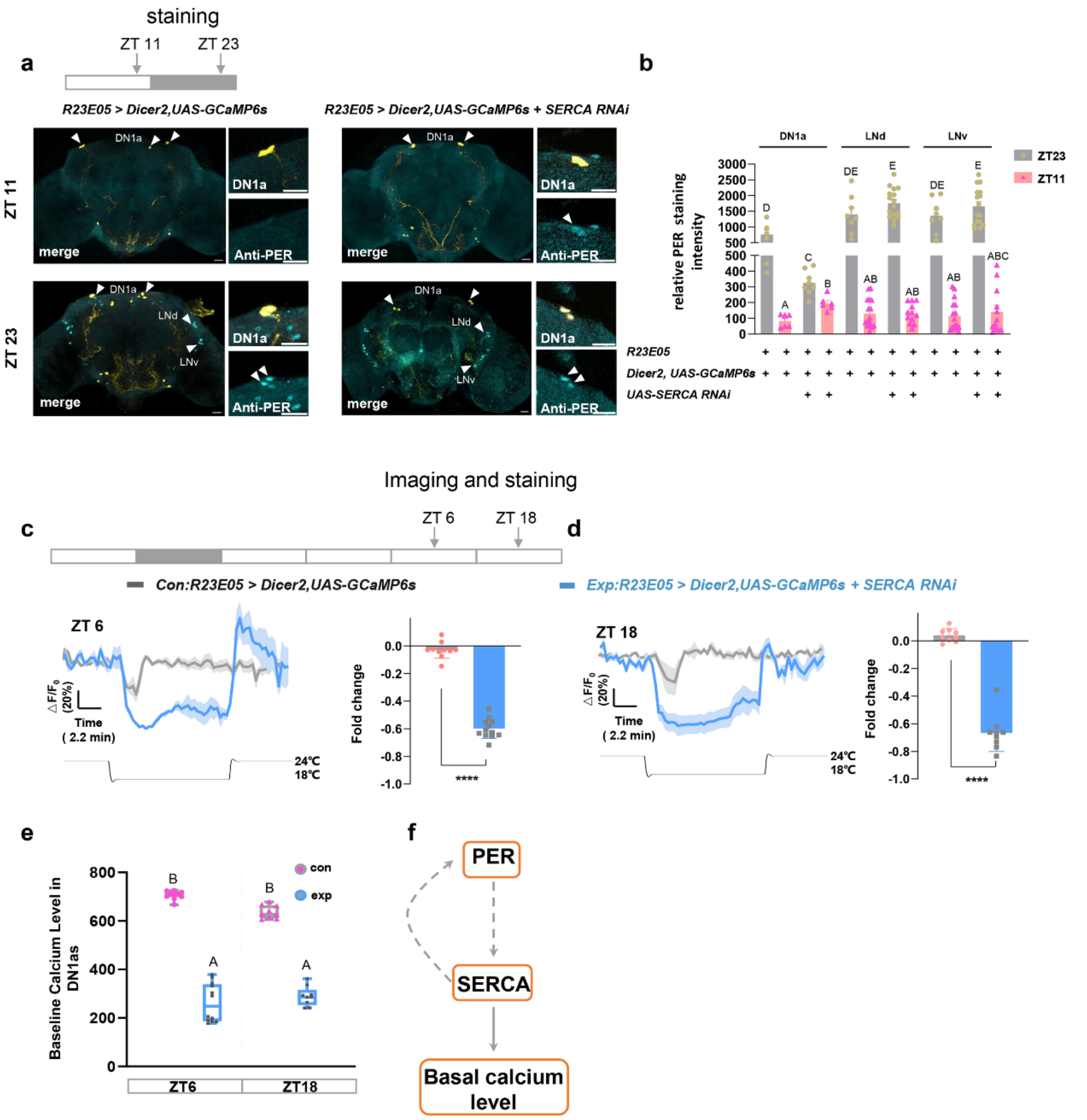

Supplementary Fig. 4: The circadian clock regulates calcium levels in DN1as in response through SERCA.

a PER staining in the brains of *R23E05 > Dicer2, UAS-GCaMP6s* (left) and *R23E05 > Dicer2, UAS-GCaMP6s + SERCA RNAi* flies (right) at ZT11 and ZT 23. Scale bars, 20 µm. The top panel indicates the time of staining.

b Quantification of PER level in DN1as, LNd and LNv (**a**, n = 6).

c,d The representative GCaMP traces ($\Delta F/F_0 \pm$ SEM) of DN1as in response to cooling in flies at ZT 6 (**a**) and ZT 18 (**b**). green curve: wild type; red curve: *per* mutant; purple curve: PER rescue. Dark shades indicate lights off (night); n = 6. The top panel indicates the time of staining and imaging. Right panel: Quantification of the relative fold change of calcium activities of DN1as in left panel. paired t-test; ****p<0.0001 (c: p= 9.37368E-15; d: p= 8.52418E-11).

e Basal GCaMP6s signals in DN1as at two circadian time points in (**c-d**).

f A model illustrating circadian proteins' influence on calcium levels in circadian neurons through SERCA.

b, e For all histograms, data are presented as mean in $\Delta F/F_0$ (%) ± SEM in the histogram; one-way ANOVA; Tukey's honest significance difference test to assess the statistical significance of the mean values within each experimental group. The letters A, B, C, D and E above the histograms denote significantly different means within each of the two groups, $p<0.05$. Specific p-values corresponding to this figure are reported in the Source Data. The baseline calcium level represents the spontaneous calcium level of DN1a before temperature fluctuations. n=6.

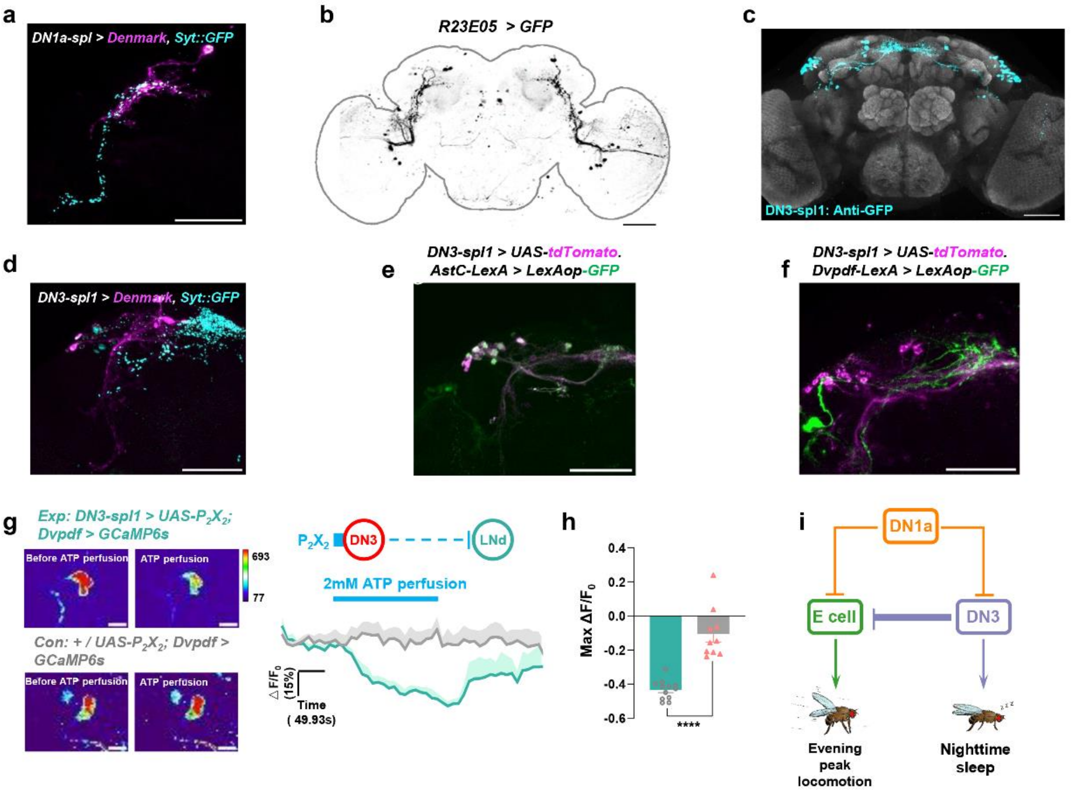

Supplementary Fig. 5: The morphological features and functional connections of DN3s at high resolution.

a, d Brains of flies co-expressing *UAS-DenMark* and *UAS-syt-GFP* reporters in DN1as (**a**) and DN3s (**d**), stained with anti-DsRed (red, postsynaptic) and anti-GFP (cyan, presynaptic). Scale bars, 50 μm.

b, c Expression pattern of the DN1a (**b**) and DN3s (**c**) driven by *R23E05-GAL4* and *DN3-spl* in the brain revealed by anti-GFP (cyan) and anti-NC82 (grey). Scale bar, 50 μm.

e, f Double staining of GFP and RFP in *AstC-LexA > LexAop-GFP, DN3-spl > UAS-tdTomato* (**e**) and *Dvpdf-LexA > LexAop-GFP, DN3-spl > UAS-tdTomato* (**f**) brain. Scale bar, 50 μm.

g Representative pseudocolored images of calcium responses of LNds before and after ATP perfusion activation of DN3s (left). Representative GCaMP traces ($\Delta F/F$) of LNds in response to DN3s activation (right, n=12). Scale bars, 10 μm.

h Quantification of relative max calcium activities of LNds in (**f**). Data are presented as mean in $\text{Max}\Delta F/F_0$ (%) ± SEM in the histogram; 2-tailed *t*-test; **p < 0.01, p= 7.00855E-07. n=12.

i Model of three inhibitory DN1as-DN3s, DN1as-LNds and DN3s-LNds circuit based on functional imaging data.

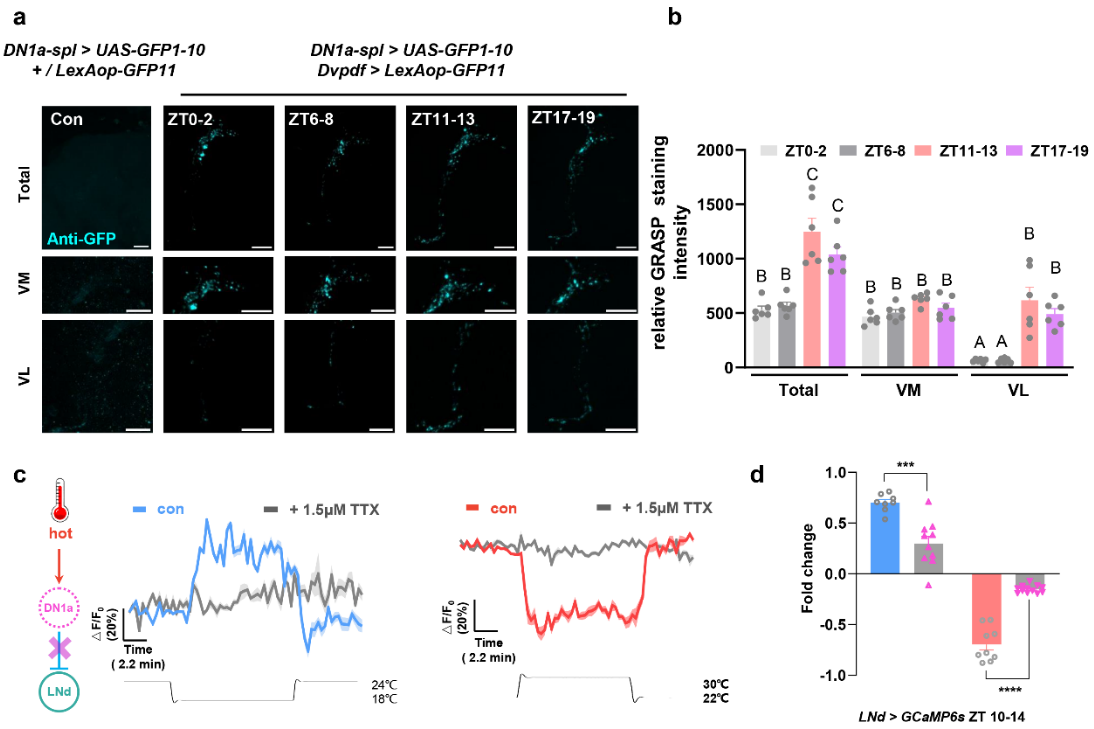

Supplementary Fig. 6: Characterization of the DN1a-LNd inhibitory circuit.

a GRASP signaling reveals plastic connections between DN1a and LNd, indicative of pronounced precept plasticity. The cyan signals of VL area indicate the presence of GRASP signaling between DN1as and LNds. VL: ventrolateral; VM: ventromedial. Scale bars, 20 μm

b Quantification of the GRASP fluorescence between DN1a and LNd. data are presented as mean in $\Delta F/F_0$ (%) ± SEM in the histogram; one-way ANOVA; Tukey's honest significance difference test to assess the statistical significance of the mean values within each experimental group. The letters A, B, C, D and E above the histograms denote significantly different means within each of the two groups, $p<0.05$. Specific p-values corresponding to this figure are reported in the Source Data. n=6.

c left: representative GCaMP traces (ΔF/F0) of LNds in response to cooling in the intact fly (blue, n=8) and adding TTX (grey, n=8). Right: Representative GCaMP traces (ΔF/F0) of LNds in response to heating in the intact fly (red, n=8) and adding TTX (grey, n=8).

d Quantification of the relative fold change of calcium activities of LNds in (**c**). Data are presented as mean in ΔF/F0 (%) ± SEM in the histogram; 2-tailed *t*-test; ****$p< 0.0001$, left panel: p= 0.0002; right panel: p= 1.15887E-09. n=8.

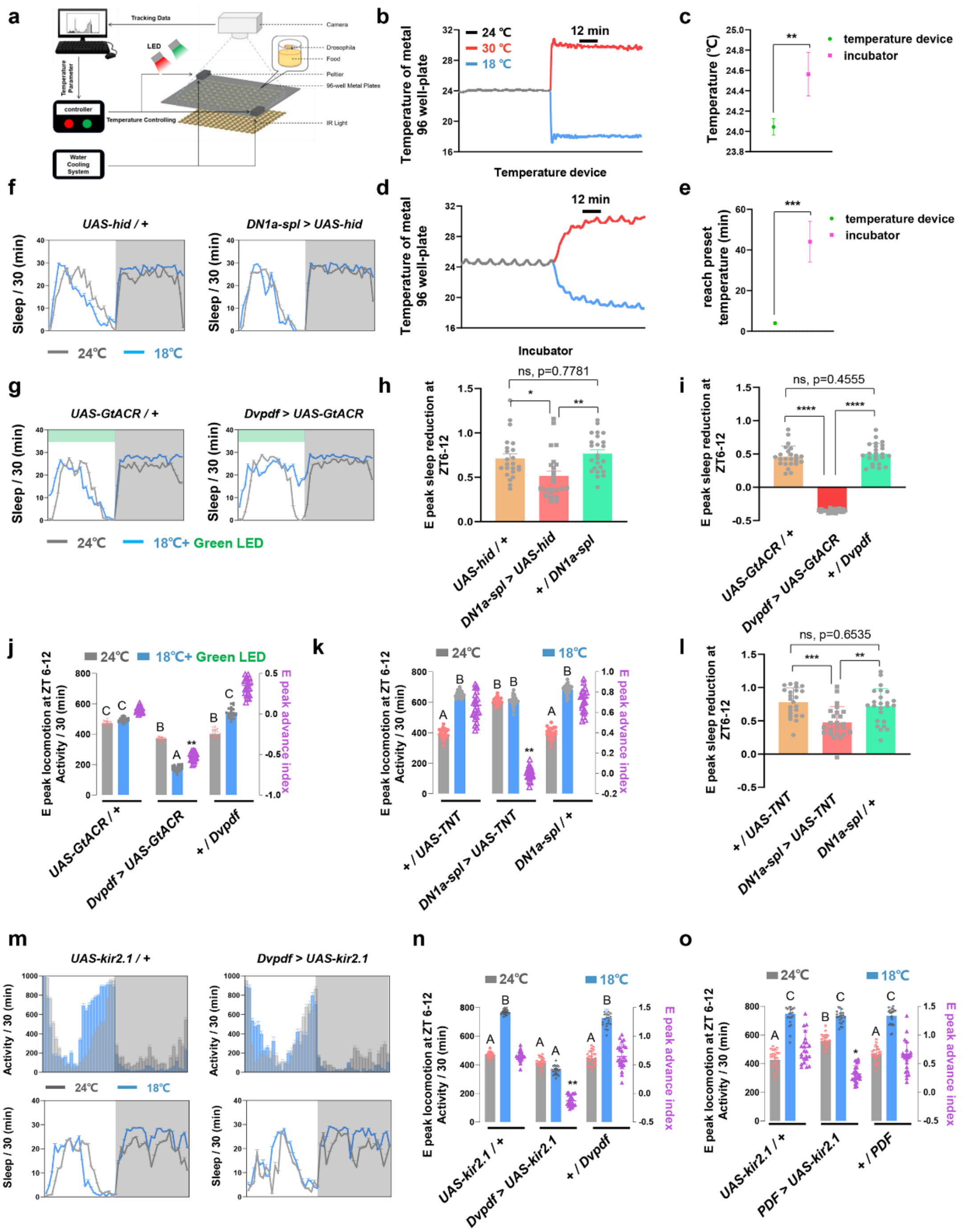

Supplementary Fig. 7: Temperature calibration of the behavioral recording system and analysis of temperature-induced sleep changes regulated by DN1a-LNd circuit.

a Schematic of the temperature-control system coupled with a sleep recording device for monitoring *Drosophila* movement.

b-e A comparison of temperature rise and fall between an incubator and a temperature control equipment was conducted. The set-points of the incubator or temperature-controlling system were changed from 24°C to 30°C (red line) and from 24°C to 18°C (blue line), respectively, to

deliver heating and cooling stimuli (refer to Methods section for details). 2-tailed *t*-test; **p < 0.01; ***p < 0.001.

f, g Representative sleep profiles of control (n=24) and DN1a-ablated (n=24) flies at 24°C (grey column) and 18°C (blue column). sleep summed in 30-min bins were plotted. Bar graphs are averages ± SEM; Dark shades indicate lights off (night). **f** related to **Fig.5g**. **g** related to **Fig.5h**.

h, i Quantification of E peak sleep reduction index in (**f and g**).

j, k Quantification of E peak locomotion (colum) and E peak advance index (magenta plot) in experimental (n=24, **j**; n=24, **k**) and control groups (+ / *UAS-TNT*) at 18°C (blue) and 24°C (grey). n=24.

l Quantification of E peak sleep reduction index in (**k**). n=24.

m, Representative locomotor activity profiles of control (n=24) and DN1a-ablated (n=24) flies at 24°C (grey column) and 18°C (blue column). activity summed in 30-min bins were plotted. Bar graphs are averages ± SEM; Dark shades indicate lights off (night).

n, o Quantification of E peak locomotion (colum) and E peak advance index (magenta) in (**n**) and PDF inhibited (**o**). The grey and blue column indicates flies'locomotion at 24°C and 18°C; the purple plots indicate E peak advance. n=24.

h, i, j, k, l, n, o For all histograms, data are presented as mean in $\Delta F/F_0$ (%) ± SEM; Statistical analysis was conducted using One-Way ANOVA followed by Tukey post-test for multiple comparisons. The letters A, B, C and D above the histograms denote significantly different means within each group. Specific p-values corresponding to this figure are reported in the Source Data. *p<0.05; **p<0.01; ****p<0.0001. ns, no significant difference.

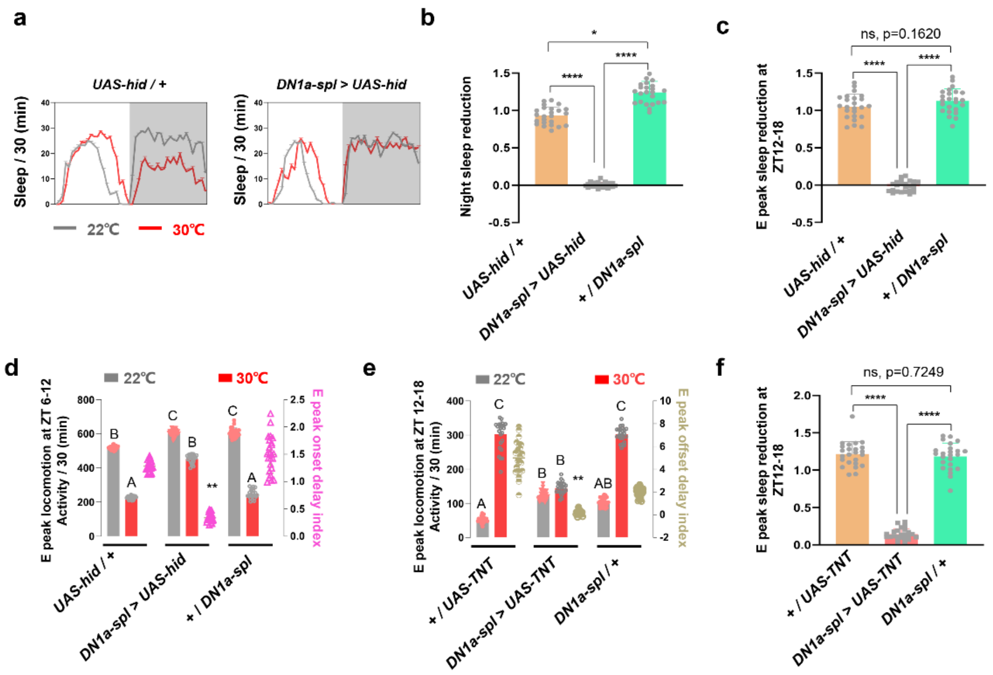

Supplementary Fig. 8: DN1a modulates hot-induced nocturnal activity changes.

a Representative sleep profiles of control (n=24) and DN1a-ablated (n=24) flies at 22℃ (grey column) and 30℃ (red column). sleep summed in 30-min bins were plotted. Bar graphs are averages ± SEM; Dark shades indicate lights off (night).

b, c Quantification of nighttime sleep reduction index (**b**) and E peak sleep reduction index (**c**) in (**a**). one-way ANOVA; Tukey's honest significance difference test to assess the statistical significance of the mean values within each experimental group. *p<0.05; ****p<0.0001. ns, no significant difference. n=24.

d, e Quantification of E peak advance index (magenta plot) and E peak offset delay index (brown plot) in experimental (DN1a ablated, n=24, **d**; *DN1a-spl* > *UAS-TNT*, n=24, **e**) and control groups (+ / *UAS-TNT*) at 22℃ (grey) and 30℃ (red). Tukey's honest significance difference test to assess the statistical significance of the mean values within each experimental group. In the provided histograms labeled A, B and C, the use of the same letter denotes the absence of a significant difference between the two groups, whereas differing letters signify a p<0.05, indicating a significant distinction between the groups. **p< 0.01.

f Quantification of E peak sleep reduction index in (**e**). one-way ANOVA; Tukey's honest significance difference test to assess the statistical significance of the mean values within each experimental group. ****p<0.0001. ns, no significant difference. n=24.

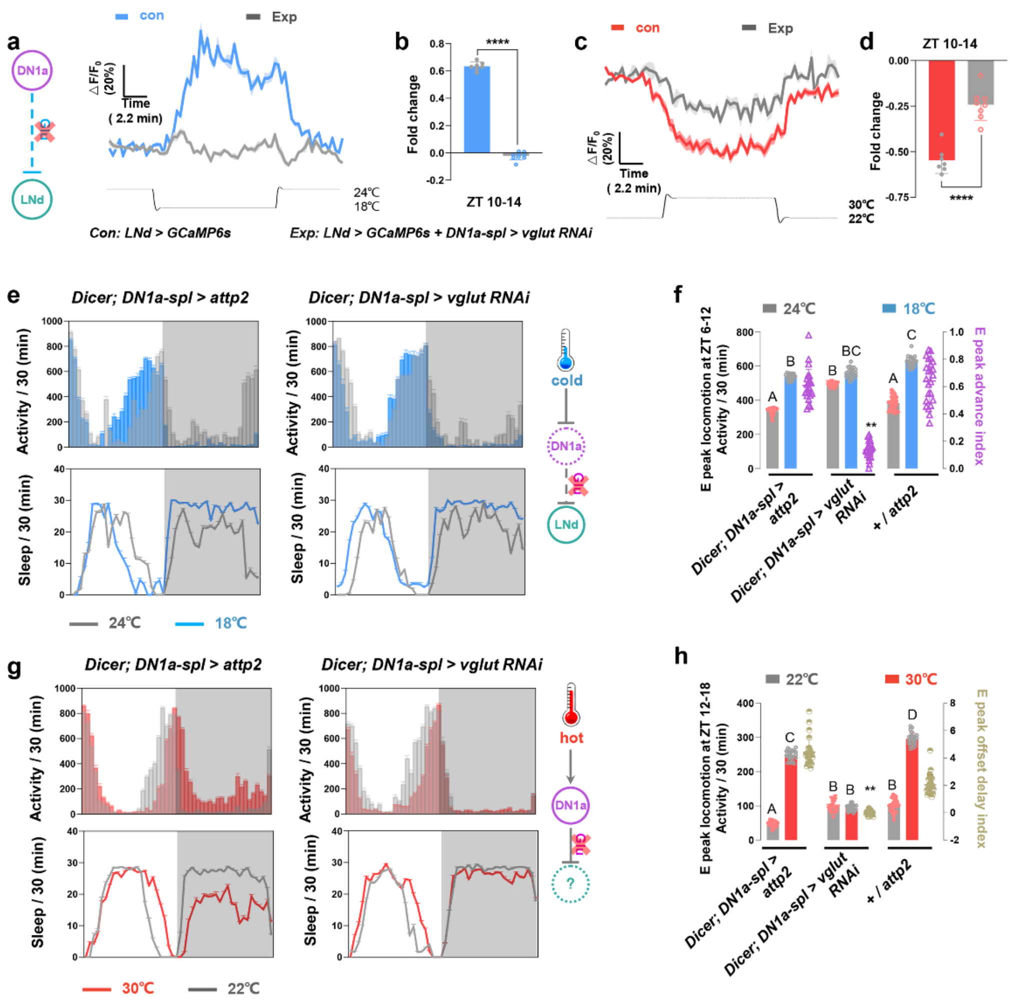

Supplementary Fig. 9: Exploring the effect of blocking glutamate release in DN1as on cold-induced E peak advance.

a, c Representative GCaMP traces ($\Delta F/F_0$) of LNds in response to cooling (**a**) and heating (**c**) in the intact fly (black, n = 16) and DN1a glutamate-silenced flies (blue or red n = 16).

b, d Quantification of the relative fold change of calcium activities of LNds in (**a**) and (**c**) respectively. Data are presented as mean in $\Delta F/F_0$ (%) ± SEM in the histogram; 2-tailed *t*-test; ****p < 0.0001, **b**: p= 2.01036E-16; **d**: p= 5.34339E-06. n=16.

e, g Representative locomotor activity profile (top panel) and sleep profile (bottom panel) of control (*DN1a-spl* > *attp2*) and experimental (*DN1a-spl* > *vglut RNAi*) flies at cooling (18°C, blue; 24°C, grey; **e**) and heating (30°C, red; 22°C, grey; **g**). Activity and sleep were quantified in 30-min bins on 1 consecutive day per condition. Data plots represent sleep (bottom) and

activity bar graphs (top) and are mean ± SEM across days and across individual flies; Dark shades indicate lights off (night). n=24.

f, h Quantification of E peak locomotion and E peak advance (magenta) / offset delay index (brown) in (**e** and **g**) with cooling (18°C, blue; 24°C, grey; **f**) and heating (30°C, red; 22°C, grey; **h**). Data are presented as mean in $\Delta F/F_0$ (%) ± SEM in the histogram; one-way ANOVA; Tukey's honest significance difference test to assess the statistical significance of the mean values within each experimental group. The letters A, B, C and D above the histograms denote significantly different means within each of the two groups, $p<0.05$. Specific p-values corresponding to this figure are reported in the Source Data. n=24.

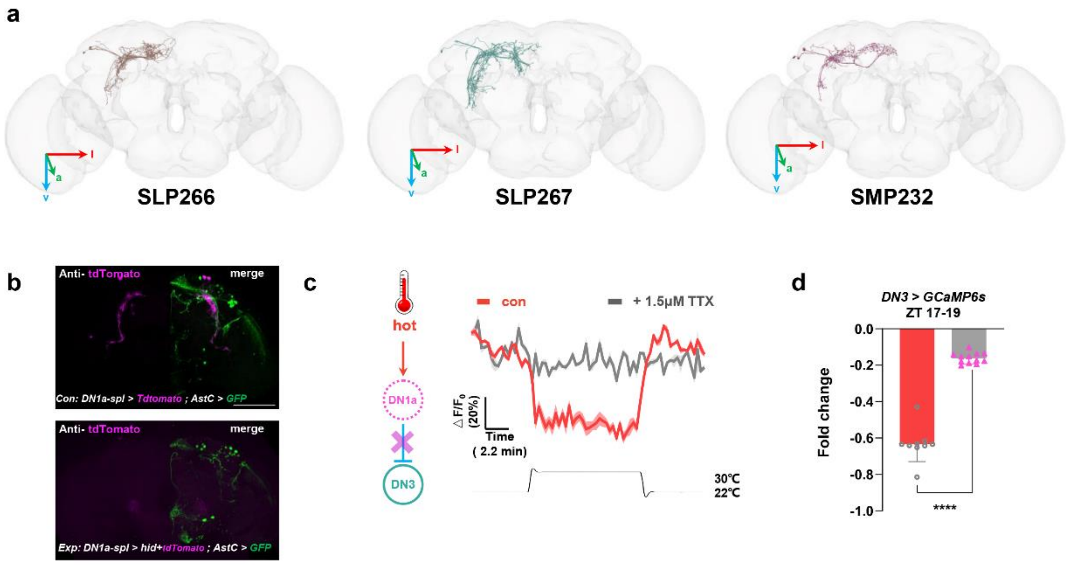

Supplementary Fig. 10: DN3 subset morphology and temperature response to TTX blockade of neuronal transmission

a, Electron microscope diagram depicting DN3-like neurons (slp266, slp267, and smp232)

b Representative double staining images of Anti-GFP and Anti-tdTomato in fly brains of *DN1a-spl* > *Tdtomato* + *AstC* > *GFP* (top panel) and *DN1a-spl* > *hid* + *AstC* > *GFP* (bottom panel). The experimental and control brains were stained with anti-GFP (green) and anti-tdTomato (magenta); Scale bars, 50 µm.

c representative GCaMP traces (ΔF/F0) of DN3s in response to heating in the intact fly (red, n=8) and adding TTX (grey, n=8).

d Quantification of the relative fold change of calcium activities of DN3s in (**e**). Data are presented as mean in ΔF/F0 (%) ± SEM in the histogram; 2-tailed *t*-test; ****p < 0.0001, p= 1.7533E-12. n=8.

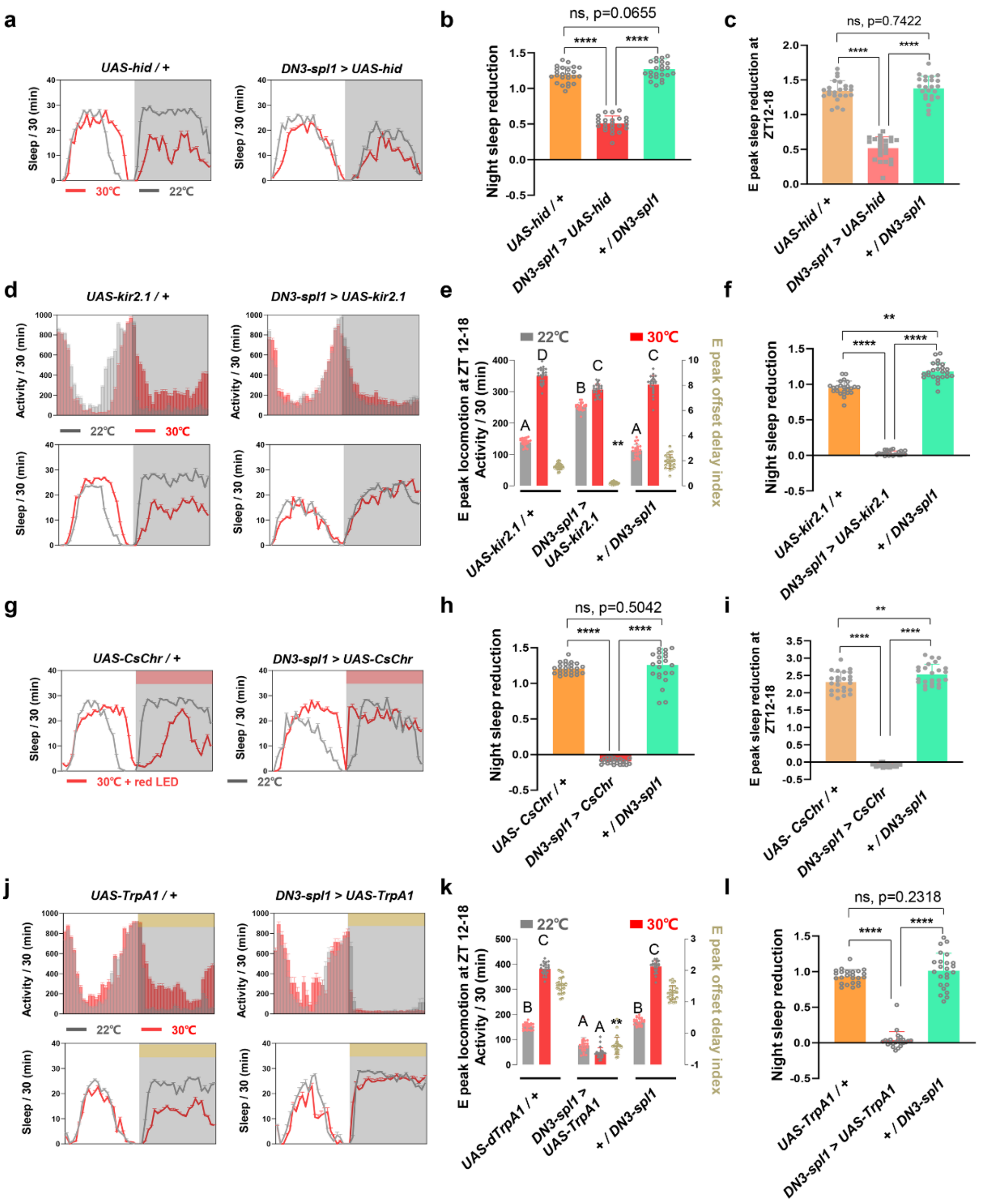

Supplementary Fig. 11: Temperature-dependent responses and functional analysis of DN3s.

a, g Representative sleep profiles of control (n=24), DN3-ablated (**a**, n=24) and optogenetic activation DN3 (**g**, n=24) flies at 22°C (grey column) and 30°C (red column).

b, h Quantification of nighttime sleep reduction index in (**a** and **g**). *p<0.05; ****p<0.0001. ns, no significant difference. **b** related to **a**. **h** related to **g**. n=24.

c, f, l Quantification of E peak sleep reduction index in (**a**, **d** and **j**). *p<0.05; ****p<0.0001. ns, no significant difference. **b** related to **a**. **e** related to **d**. **l** related to **j.** n=24.

d, j Representative locomotor activity and sleep profiles of control (n=24), DN3 inhibited groups (*DN3-spl > UAS-kir2.1*, **d**, n=24.) and DN3 activation groups (*DN3-spl > UAS-dTrpA1,* **j**, n=24). flies at 22°C (grey column) and 30°C (red column).

a, g, d, j Sleep and activity summed in 30-min bins were plotted. Bar graphs are averages ± SEM; Dark shades indicate lights off (night). yellow shades indicate hot temperature coditions (30°C). n=24.

e, k Quantification of E peak locomotion (colum) and E peak offset delay index (brown) in (**d** and **j**) with hot conditions (30°C, red). Data are presented as mean in $\Delta F/F_0$ (%) ± SEM in the histogram; one-way ANOVA; Tukey's honest significance difference test to assess the statistical significance of the mean values within each experimental group. The letters A, B, C and D above the histograms denote significantly different means within each of the two groups, $p<0.05$. Specific p-values corresponding to this figure are reported in the Source Data. **$p<0.01$. **e** related to **d**. **k** related to **j**. n=24.

b, c, f, h, one-way ANOVA; Tukey's honest significance difference test to assess the statistical significance of the mean values within each experimental group.

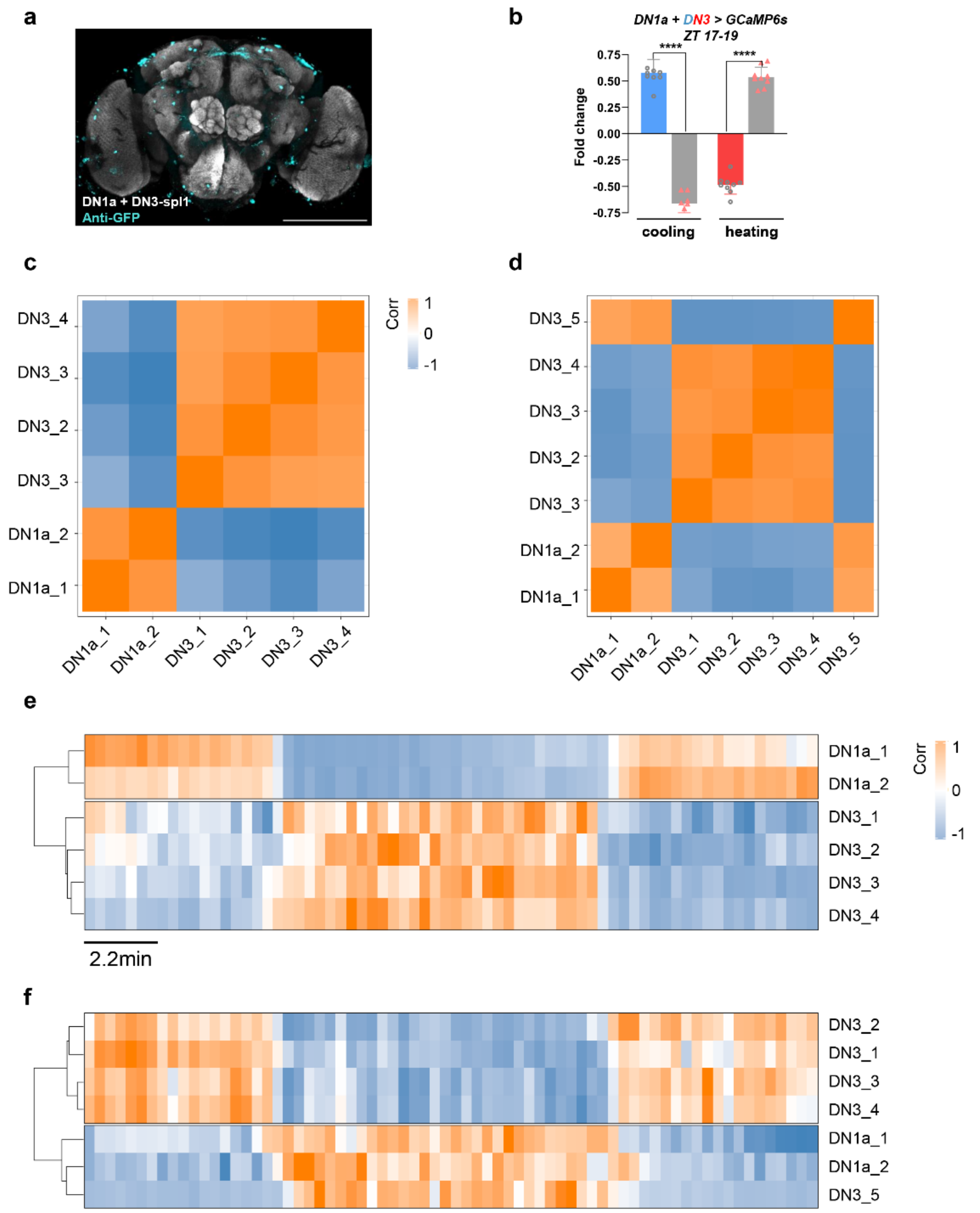

Supplementary Fig. 12: A schematic representation of the morphology of DN3s.

a Expression pattern of the DN1as driven by *DN1a + DN3-spl* in the brain revealed by anti-GFP (cyan) and anti-NC82 (grey). Scale bar, 50 μm.

b Quantification of the relative fold change of calcium activities of DN1as (grey) and DN3s (blue and red) in (**Fig. 7a, c**). Data are presented as mean in $\Delta F/F_0$ (%) ± SEM in the histogram; 2-tailed *t*-test; ns: no significantly difference; ****$p < 0.0001$, left: p= 4.98912E-13; right: p= 7.84438E-14. n=8.

c-d Pairwise correlation matrices of DNas and DN3s in response to cooling (**c**) and heating (**d**). related to **Supplementary Fig. 7a, c**.

e-f Pairwise correlation matrices of DNas and DN3s real-time calcium activity in response to cooling (**e**) and heating (**f**). related to **Supplementary Fig. 7a, c**.

Supplementary Videos

Supplementary Video. 1: The representative video showing a tethered experimental fly expressing GCaMP with *Clk856-GAL4* on an air-supported ball during cooling, related to (Fig. 1c-e).

Top left: the locomotion of fly; top right: the reaction to cooling in circadian neurons' calcium (*Clk856-GAL4*) activity; bottom left: the velocity of a fly's reaction to cooling; bottom right: the reaction to cooling in the DN1as' calcium activity. White arrow indicates DN1as. Color rendering of video screens through Fiji to enhance neuronal calcium activity visualization.

Supplementary Video. 2: The representative video showing a tethered experimental fly expressing GCaMP with *Clk856-GAL4* on an air-supported ball during heating, related to (Fig. 1f-h).

Top left: the locomotion of fly; top right: the reaction to heating in circadian neurons' calcium (*Clk856-GAL4*) activity; bottom left: the velocity of a fly's reaction to heating; bottom right: the reaction to heating in the DN1as' calcium activity. White arrow indicates DN1as. Color rendering of video screens through Fiji to enhance neuronal calcium activity visualization.

Supplementary Video. 3: The 3D brain reconstruction shows SMP232 neurons labeled downstream of DN1a via restricted *trans-Tango*. Related to (Fig. 4b-c). Color rendering of video screens through Fiji to enhance neuronal calcium activity visualization.

Supplementary Video. 4: The representative video showing a tethered experimental fly expressing GCaMP with *DN1a* + *DN3-spl* on an air-supported ball during cooling, related to (Fig. 7a-b).

Top left: the locomotion of fly; top right: the reaction to heating in the circadian neurons' calcium (*DN1a* + *DN3-spl*) activity; lower left: the velocity of a fly's reaction to cooling; lower right: the reaction to cooling in the DN1as and DN3s' calcium activity. Color rendering of video screens through Fiji to enhance neuronal calcium activity visualization.

Supplementary Video. 5: The representative video showing a tethered experimental fly expressing GCaMP with *DN1a + DN3-spl* on an air-supported ball during heating, related to (Fig. 7c-d).

Top left: the locomotion of fly; top right: the reaction to heating in the circadian neurons' calcium (*DN1a + DN3-spl*) activity; lower left: the velocity of a *Drosophila's* reaction to heating; lower right: the reaction to heating in the DN1as and DN3s' calcium activity. Color rendering of video screens through Fiji to enhance neuronal calcium activity visualization.

Supplementary Table 1: Genotypes of the flies used in this study, experimental model and subject details.

**Fig #**	**Genotype**
1a, c-h	*Clk856-GAL4, UAS-IVS-GCaMP6s*
1i	*R14F03-GAL4.AD / UAS-tdTomato; VT002963-GAL4.DBD / +*
1j	*R14F03-GAL4.AD / UAS-IVS-GCaMP6s; VT002963-GAL4.DBD / +*
2a	*R14F03-GAL4.AD / UAS-tdTomato; VT002963-GAL4.DBD / UAS-IVS-GCaMP7s*
2c	*R14F03-GAL4.AD / UAS-IVS-GCaMP6s; VT002963-GAL4.DBD / +*
2e-f	*R14F03-GAL4.AD / UAS-IVS-GCaMP6s; VT002963-GAL4.DBD / +*
2e-f	*per0; R14F03-GAL4.AD / UAS-IVS-GCaMP6s; VT002963-GAL4.DBD / +*
2e-f	*per0; R14F03-GAL4.AD / UAS-IVS-GCaMP6s; VT002963-GAL4.DBD / UAS-PER*
3a	*R14F03-GAL4.AD / UAS-Stinger-GFP, LexAop-tdTomato; VT002963-GAL4.DBD / vglut-LexA*
3c-d	*R14F03-GAL4.AD / trans-Tango, QUAS-GFP; VT002963-GAL4.DBD / UAS-tdTomato*
3e	*R14F03-GAL4.AD / Dvpdf-LexA; VT002963-GAL4.DBD / UAS-tdTomato, LexAop-GFP*
3f	*R14F03-GAL4.AD / UAS-P2X2, Dvpdf-LexA; VT002963-GAL4.DBD / LexAop-GCaMP7s*
4a	*R14F03-GAL4.AD / trans-Tango, QUAS-GCaMP3; VT002963-GAL4.DBD / UAS-tdTomato*
4b-c	*AstC-LexA / trans-Tango, QUAS-FLP; R23E05-GAL4 / LexAop-FRT-RFP-Stop-FRT- GFP*
4d	*AstC-LexA /LexAop-FLP; GluClα-GAL4 / UAS-FRT-STOP-FRT-CsChrimson. mVenus*
4e	*R14F03-GAL4.AD / AstC-LexA; VT002963-GAL4.DBD / UAS-tdTomato,*

	LexAop-GFP
4f	*R14F03-GAL4.AD / AstC-LexA, LexAop-GCaMP6s; VT002963-GAL4.DBD / UAS-*P_2X_2
5b	*Dvpdf-LexA; LexAop-GCaMP7s*
5d, e	*UAS-hid; R14F03-GAL4.AD / Dvpdf-LexA; VT002963-GAL4.DBD / UAS-tdTomato, LexAop-GCaMP6s*
5d	*UAS-hid; + / Dvpdf-LexA; + / UAS-tdTomato, LexAop-GCaMP6s*
5g	*UAS-hid; R14F03-GAL4.AD / +; VT002963-GAL4.DBD / +*
5h	*UAS-GtACR / Dvpdf-GAL4*
5j	*UAS-hid; R14F03-GAL4.AD / +; VT002963-GAL4.DBD / +*
6b-c	*AstC-LexA; LexAop-GCaMP7s*
6d	*UAS-hid; R14F03-GAL4.AD / AstC-LexA; VT002963-GAL4.DBD / UAS-tdTomato, LexAop-GCaMP6s*
6d	*UAS-hid; + / AstC-LexA; + / UAS-tdTomato, LexAop-GCaMP6s*
6f	*AstC-LexA; LexAop-GCaMP7s*
6h	*UAS-hid; R67F03-GAL4.AD / +; VT002670-GAL4.DBD / +*
6i	*R67F03-GAL4.AD / UAS-CsChrimson; VT002670-GAL4.DBD / +*
7a, c	*R67F03-GAL4.AD / UAS-GCaMP6s; VT002670-GAL4.DBD / R23E05-GAL4*
**Supplementary Fig #**	
1b	*R14F03-GAL4.AD / R43D05-LexA; VT002963-GAL4.DBD / UAS-tdTomato, LexAop-GFP*
1c	*R14F03-GAL4.AD / R18H11-LexA; VT002963-GAL4.DBD / UAS-tdTomato, LexAop-GFP*
1d	*UAS-RFP, LexAop-GCaMP6s; R14F03-GAL4.AD / +; VT002963-GAL4.DBD / Clk4.1m-LexA*
1g-h	*R14F03-GAL4.AD / UAS-GFP; VT002963-GAL4.DBD / +*
1g-h	*R14F03-GAL4.AD / UAS-IVS-GCaMP6s; VT002963-GAL4.DBD / +*
1j-i	*Clk856-GAL4, UAS-IVS-GCaMP6s*

2a-b	*R14F03-GAL4.AD / UAS-IVS-GCaMP6s; VT002963-GAL4.DBD / +*
2c-d	*R60H12 -GAL4.AD / UAS-IVS-GCaMP6s; VT032805 -GAL4.DBD / +*
2f	*R14F03-GAL4.AD / UAS-IVS-GCaMP6s; VT002963-GAL4.DBD / +*
2g	*per0; R14F03-GAL4.AD / UAS-IVS-GCaMP6s; VT002963-GAL4.DBD / UAS-PER*
2i-j	*R14F03-GAL4.AD / UAS-IVS-GCaMP6s; VT002963-GAL4.DBD / +*
2i-j	*per0; R14F03-GAL4.AD / UAS-IVS-GCaMP6s; VT002963-GAL4.DBD / +*
2i-j	*per0; R14F03-GAL4.AD / UAS-IVS-GCaMP6s; VT002963-GAL4.DBD / UAS-PER*
3a-b	*dicer2; UAS-IVS-GCaMP6s/+; R23E05-GAL4/+*
3a-b	*dicer2; UAS-IVS-GCaMP6s/+; R23E05-GAL4/UAS-SK DN*
3a-b	*dicer2; UAS-IVS-GCaMP6s/+; R23E05-GAL4/UAS-SERCA RNAi*
3a-b	*dicer2; UAS-IVS-GCaMP6s/+; R23E05-GAL4/UAS-*Na^{har} *RNAi*
3e	*dicer2; +/+; R23E05-GAL4/attp2*
3e	*dicer2; +/+; R23E05-GAL4/UAS-SK DN*
3e	*dicer2; +/+; R23E05-GAL4/UAS-SercaRNAi*
3e	*dicer2; +/+; R23E05-GAL4/UAS-*Na^{har} *RNAi*
3e	*+/+; +/attp2*
4a, c	*dicer2; UAS-IVS-GCaMP6s/+; R23E05-GAL4/+*
4a, d	*dicer2; UAS-IVS-GCaMP6s/+; R23E05-GAL4/UAS-SERCA RNAi*
5a	*R14F03-GAL4.AD / UAS-Denmark, UAS-Syt::GFP; VT002963-GAL4.DBD / +*
5b	*UAS-GFP; R23E05-GAL4*
5c	*R67F03-GAL4.AD / UAS-GFP; VT002670-GAL4.DBD / +*
5d	*R67F03-GAL4.AD / UAS-Denmark, UAS-Syt::GFP; VT002670-GAL4.DBD / +*
5e	*R67F03-GAL4.AD / AstC-LexA; VT002670-GAL4.DBD / UAS-tdTomato, LexAop-GFP*
5f	*R67F03-GAL4.AD / Dvpdf-LexA; VT002670-GAL4.DBD / UAS-tdTomato,*

	LexAop-GFP
5g	*R67F03-GAL4.AD / UAS-P2X2, Dvpdf-LexA; VT002670-GAL4.DBD / LexAop-GCaMP7s*
5g	*+ / UAS-P2X2, Dvpdf-LexA; + / LexAop-GCaMP7s*
6a	*R14F03-GAL4.AD / Dvpdf-LexA; VT002963-GAL4.DBD / UAS-GRASP*
6a	*R14F03-GAL4.AD / +; VT002963-GAL4.DBD / UAS-GRASP*
6b	*Dvpdf-LexA / +; LexAop-GCaMP6s / +*
7f	*UAS-hid; R14F03-GAL4.AD / +; VT002963-GAL4.DBD / +*
7g	*UAS-GtACR / Dvpdf-GAL4*
7k	*R14F03-GAL4.AD / +; VT002963-GAL4.DBD / UAS-TNT*
7m	*Dvpdf-GAL4 / UAS-Kir2.1; + / +*
7m	*Dvpdf-GAL4 / +; + / +*
7m	*+ / UAS-Kir2.1; + / +*
7o	*pdf-GAL4 / UAS-Kir2.1; + / +*
8a	*UAS-hid; R14F03-GAL4.AD / +; VT002963-GAL4.DBD / +*
8e	*R14F03-GAL4.AD / +; VT002963-GAL4.DBD / UAS-TNT*
9a,c	*R14F03-GAL4.AD / Dvpdf-LexA, LexAop-GCaMP6s; VT002963-GAL4.DBD / UAS-vglut RNAi*
9e,g	*Dicer2; R14F03-GAL4.AD / +; VT002963-GAL4.DBD / UAS-vglut RNAi*
10b	*UAS-hid; R14F03-GAL4.AD / AstC-LexA; VT002963-GAL4.DBD / UAS-tdTomato, LexAop-GCaMP6s*
10b	*UAS-hid; + / AstC-LexA; + / UAS-tdTomato, LexAop-GCaMP6s*
10c	*AstC-LexA / +; LexAop-GCaMP6s / +*
11a	*UAS-hid; R67F03-GAL4.AD / +; VT002670-GAL4.DBD / +*
11d	*R67F03-GAL4.AD / UAS-Kir2.1; VT002670-GAL4.DBD / +*
11g	*R67F03-GAL4.AD / UAS-CsChrimson; VT002670-GAL4.DBD / +*
11j	*R67F03-GAL4.AD / UAS-TrpA1; VT002670-GAL4.DBD / +*
12a	*R67F03-GAL4.AD / UAS-GFP; VT002670-GAL4.DBD / R23E05-GAL4*
**Supplementary**	

**Videos #**	
1-2	*Clk856-GAL4, UAS-IVS-GCaMP6s*
3	*AstC-LexA / trans-Tango, QUAS-FLP; R23E05-GAL4 / LexAop-FRT-RFP-Stop-FRT- GFP*
4-5	*R67F03-GAL4.AD / UAS-GCaMP6s; VT002670-GAL4.DBD / R23E05-GAL4*
