# Supplementary material for: Dynamic encoding of temperature in the central circadian circuit coordinates physiological activities
Source: Nat Commun. 2024 Apr 2;15:2834. doi: 10.1038/s41467-024-47278-5 (PMC10987497; doi:10.1038/s41467-024-47278-5)
Supplement: Supplementary file 3 — Description of Additional Supplementary Files [file 41467_2024_47278_MOESM3_ESM.pdf]

Description of Additional Supplementary Files

Supplementary Video. 1: The representative video showing a tethered experimental fly expressing GCaMP with *Clk856-GAL4* on an air-supported ball during cooling, related to (Fig. 1c-e).

Top left: the locomotion of fly; top right: the reaction to cooling in circadian neurons' calcium (*Clk856-GAL4*) activity; bottom left: the velocity of a fly's reaction to cooling; bottom right: the reaction to cooling in the DN1as' calcium activity. White arrow indicates DN1as. Color rendering of video screens through Fiji to enhance neuronal calcium activity visualization.

Supplementary Video. 2: The representative video showing a tethered experimental fly expressing GCaMP with *Clk856-GAL4* on an air-supported ball during heating, related to (Fig. 1f-h).

Top left: the locomotion of fly; top right: the reaction to heating in circadian neurons' calcium (*Clk856-GAL4*) activity; bottom left: the velocity of a fly's reaction to heating; bottom right: the reaction to heating in the DN1as' calcium activity. White arrow indicates DN1as. Color rendering of video screens through Fiji to enhance neuronal calcium activity visualization.

Supplementary Video. 3: The 3D brain reconstruction shows SMP232 neurons labeled downstream of DN1a via restricted *trans-Tango*. Related to (Fig. 4b-c). Color rendering of video screens through Fiji to enhance neuronal calcium activity visualization.

Supplementary Video. 4: The representative video showing a tethered experimental fly expressing GCaMP with *DN1a* + *DN3-spl* on an air-supported ball during cooling, related to (Fig. 7a-b).

Top left: the locomotion of fly; top right: the reaction to heating in the circadian neurons' calcium (*DN1a* + *DN3-spl*) activity; lower left: the velocity of a fly's reaction to cooling; lower right: the reaction to cooling in the DN1as and DN3s' calcium activity. Color rendering of video screens through Fiji to enhance neuronal calcium activity visualization.

Supplementary Video. 5: The representative video showing a tethered experimental fly expressing GCaMP with *DN1a* + *DN3-spl* on an air-supported ball during heating, related to (Fig. 7c-d).

Top left: the locomotion of fly; top right: the reaction to heating in the circadian neurons' calcium (*DN1a* + *DN3-spl*) activity; lower left: the velocity of a *Drosophila's* reaction to heating; lower right: the reaction to heating in the DN1as and DN3s' calcium activity. Color rendering of video screens through Fiji to enhance neuronal calcium activity visualization.
