# Supplementary material for: Dynamic encoding of temperature in the central circadian circuit coordinates physiological activities
Source: Nat Commun. 2024 Apr 2;15:2834. doi: 10.1038/s41467-024-47278-5 (PMC10987497; doi:10.1038/s41467-024-47278-5)
Supplement: Supplementary file 9 — Reporting Summary [file 41467_2024_47278_MOESM9_ESM.pdf]

nature portfolio

Corresponding author(s): Fang Guo

Last updated by author(s): Mar 12, 2024

Reporting Summary

Nature Portfolio wishes to improve the reproducibility of the work that we publish. This form provides structure for consistency and transparency in reporting. For further information on Nature Portfolio policies, see our Editorial Policies and the Editorial Policy Checklist.

Statistics

For all statistical analyses, confirm that the following items are present in the figure legend, table legend, main text, or Methods section.

n/a	Confirmed	
☐	☒	The exact sample size (*n*) for each experimental group/condition, given as a discrete number and unit of measurement
☐	☒	A statement on whether measurements were taken from distinct samples or whether the same sample was measured repeatedly
☐	☒	The statistical test(s) used AND whether they are one- or two-sided<br>*Only common tests should be described solely by name; describe more complex techniques in the Methods section.*
☐	☒	A description of all covariates tested
☐	☒	A description of any assumptions or corrections, such as tests of normality and adjustment for multiple comparisons
☐	☒	A full description of the statistical parameters including central tendency (e.g. means) or other basic estimates (e.g. regression coefficient) AND variation (e.g. standard deviation) or associated estimates of uncertainty (e.g. confidence intervals)
☐	☒	For null hypothesis testing, the test statistic (e.g. *F*, *t*, *r*) with confidence intervals, effect sizes, degrees of freedom and *P* value noted<br>*Give P values as exact values whenever suitable.*
☒	☐	For Bayesian analysis, information on the choice of priors and Markov chain Monte Carlo settings
☒	☐	For hierarchical and complex designs, identification of the appropriate level for tests and full reporting of outcomes
☒	☐	Estimates of effect sizes (e.g. Cohen's *d*, Pearson's *r*), indicating how they were calculated

Our web collection on statistics for biologists contains articles on many of the points above.

Software and code

Policy information about availability of computer code

Data collection	Two-photon and confocal images were processed using Fiji. In-vivo imaging experiments utilized an upright Olympus FV1200MPE microscope equipped with a 40×0.9 numerical aperture water immersion objective, capturing images at a resolution of 512 pixels × 256 pixels using FV10-ASW (Ver.03.01). Behavioral assays recorded movements with a C930s Pro HD Webcam - Logitech.
Data analysis	The calcium signal data analysis was performed using ImageJ2/FIJI.<br>The speed of changes in supported balls was analyzed using Fitrac.<br>The sleep analysis of fruit flies was conducted using Pysolo and MATLAB.<br>Statistical difference analysis of data was performed using SPSS and Graphad software.

For manuscripts utilizing custom algorithms or software that are central to the research but not yet described in published literature, software must be made available to editors and reviewers. We strongly encourage code deposition in a community repository (e.g. GitHub). See the Nature Portfolio guidelines for submitting code & software for further information.

Data

Policy information about availability of data

All manuscripts must include a data availability statement. This statement should provide the following information, where applicable:

- Accession codes, unique identifiers, or web links for publicly available datasets
- A description of any restrictions on data availability
- For clinical datasets or third party data, please ensure that the statement adheres to our policy

The data availability statement provided in the manuscript includes the required information. The data generated during the current study are available as a source data file and can be obtained from the corresponding author upon request. Additionally, the following databases were utilized in this study: Flylight (https://www.janelia.org/project-team/flylight), Vienna Drosophila Resource Center (https://shop.vbc.ac.at/vdrc_store/), Codex (https://codex.flywire.ai/), Bloomington Drosophila Stock Center (https://bdsc.indiana.edu/) and neuPrint (https://neuprint.janelia.org/). Custom code used in the study is available through the GallioLab GitHub repository (https://github.com/lihailiang7794)

Research involving human participants, their data, or biological material

Policy information about studies with human participants or human data. See also policy information about sex, gender (identity/presentation), and sexual orientation and race, ethnicity and racism.

Reporting on sex and gender	N/A
Reporting on race, ethnicity, or other socially relevant groupings	N/A
Population characteristics	N/A
Recruitment	N/A
Ethics oversight	N/A

Note that full information on the approval of the study protocol must also be provided in the manuscript.

Field-specific reporting

Please select the one below that is the best fit for your research. If you are not sure, read the appropriate sections before making your selection.

☒ Life sciences ☐ Behavioural & social sciences ☐ Ecological, evolutionary & environmental sciences

For a reference copy of the document with all sections, see nature.com/documents/nr-reporting-summary-flat.pdf

Life sciences study design

All studies must disclose on these points even when the disclosure is negative.

Sample size	Experiments involving quantification of calcium trace and sleep recording used n=3 as the minimal replicate number.
Data exclusions	Data were not excluded from analysis.
Replication	For the in-vivo calcium imaging experiments, repeated measurements were taken from a specified number of cells and animals, as indicated in the corresponding Figure legend panels. In all other experiments, results were replicated across datasets in different flies and were reproduced on a minimum of 3 animals per condition.
Randomization	No differential treatment was employed, thus standard randomization procedures were not applicable. Experimental genotypes and controls were identified using genetic markers as appropriate. Testing of both control and experimental groups was interspersed throughout the study to minimize potential biases.
Blinding	The experimenters were not blinded to genotypes, as blinding was deemed unnecessary due to automated data analysis, which eliminated potential operator bias

Reporting for specific materials, systems and methods

We require information from authors about some types of materials, experimental systems and methods used in many studies. Here, indicate whether each material, system or method listed is relevant to your study. If you are not sure if a list item applies to your research, read the appropriate section before selecting a response.

Materials & experimental systems

n/a	Involved in the study	
☐	☒	Antibodies
☒	☐	Eukaryotic cell lines
☒	☐	Palaeontology and archaeology
☐	☒	Animals and other organisms
☒	☐	Clinical data
☒	☐	Dual use research of concern
☒	☐	Plants

Methods

n/a	Involved in the study	
☒	☐	ChIP-seq
☒	☐	Flow cytometry
☒	☐	MRI-based neuroimaging

Antibodies

Antibodies used	Anti-Brp (mouse monoclonal) DSHB Cat# nc82;RRID: AB_2314866<br>Chicken anti-GFP Abcam AB13970; RRID:AB_300798<br>Rabbit anti-DsRed Takara Bio USA Cat# 632496; RRID: AB_10013483<br>Goat anti-Mouse IgG (H+L) Alexa Fluor 488 Thermo Fisher Scientific Cat# A-11001; RRID: AB_2534069<br>Goat anti-Mouse IgG (H+L) Alexa Fluor 635 Thermo Fisher Scientific Cat# A-31574; RRID: AB_2536184<br>Goat anti-Chicken IgY (H+L), Alexa Fluor 488 Thermo Fisher Scientific Cat# A-11039; RRID: AB_2534096<br>Goat anti-Rabbit IgG (H+L) Alexa Fluor 635 Thermo Fisher Scientific Cat# A-31577; RRID: AB_2536187<br>Rabbit anti-PER (Sun LL et al, Neuron, 2022)<br>Rabbit anti-GFP Thermo Fisher Scientific<br>Cat#G10362; RRID: AB_2536526<br>Donkey anti-Rabbit IgG (H+L) Alexa Fluor 488 Rockland Cat# 612-156-120; RRID: AB_10893386
Validation	All antibodies used in this study were previously validated in published works (Sun LL, et al. Neuron. 2022).

Animals and other research organisms

Policy information about studies involving animals; ARRIVE guidelines recommended for reporting animal research, and Sex and Gender in Research

Laboratory animals	The Table of Genotypes (Supplementary Material Table 1) furnishes a comprehensive description of the fly genotypes utilized in each experiment, along with the origin of the transgenic stocks, including references to specific figure panels. Further details on the generation of transgenic stocks developed for this study can be found in the corresponding Methods section. Additionally, specific fly age is specified in the Methods section.
Wild animals	No wild animals were used in this study.
Reporting on sex	All findings apply only to male Drosophila melanogaster.
Field-collected samples	No field-collected samples were used in this study.
Ethics oversight	The experimental subjects for this study are fruit flies and did not require ethical oversight.

Note that full information on the approval of the study protocol must also be provided in the manuscript.
